# A New Synthetic Amphiploid (AADDAA) between *Gossypium hirsutum* and *G*. *arboreum* Lays the Foundation for Transferring Resistances to Verticillium and Drought

**DOI:** 10.1371/journal.pone.0128981

**Published:** 2015-06-10

**Authors:** Yu Chen, Yingying Wang, Ting Zhao, Jianwei Yang, Shouli Feng, Wajad Nazeer, Tianzhen Zhang, Baoliang Zhou

**Affiliations:** State Key Laboratory of Crop Genetics & Germplasm Enhancement, MOE Hybrid Cotton R&D Engineering Research Center, Nanjing Agricultural University, Nanjing, People’s Republic of China; National Key Laboratory of Crop Genetic Improvement, CHINA

## Abstract

*Gossypium arboreum*, a cultivated cotton species (2n = 26, AA) native to Asia, possesses invaluable characteristics unavailable in the tetraploid cultivated cotton gene pool, such as resistance to pests and diseases and tolerance to abiotic stresses. However, it is quite difficult to transfer favorable traits into Upland cotton through conventional methods due to the cross-incompatibility of *G*. *hirsutum* (2n = 52, AADD) and *G*. *arboreum*. Here, we improved an embryo rescue technique to overcome the cross-incompatibility between these two parents for transferring favorable genes from *G*. *arboreum* into *G*. *hirsutum*. Our results indicate that MSB2K supplemented with 0.5 mgl^-1^ kinetin and 250 mg^-1^ casein hydrolysate is an efficient initial medium for rescuing early (3 d after pollination) hybrid embryos. Eight putative hybrids were successfully obtained, which were further verified and characterized by cytology, molecular markers and morphological analysis. The putative hybrids were subsequently treated with different concentrations of colchicine solution to double their chromosomes. The results demonstrate that four putative hybrid plants were successfully chromosome-doubled by treatment with 0.1% colchicine for 24 h and become amphiploid, which were confirmed by cytological observation, self-fertilization and backcrossing. Preliminary assessments of resistance at seedling stage indicate that the synthetic amphiploid showed highly resistant to Verticillium and drought. The synthetic amphiploid between *G*. *hirsutum* × *G*. *arboreum* would lay the foundation for developing *G*. *arboreum*-introgressed lines with the uniform genetic background of *G*. *hirsutum* acc TM-1, which would greatly enhance and simplify the mining, isolation, characterization, cloning and use of *G*. *arboreum*-specific desirable genes in future cotton breeding programs.

## Introduction

Upland cotton (*Gossypium hirsutum* L., 2n = 52, AADD) is the most widely cultivated cotton species, accounting for >90% of cotton lint production worldwide. The challenge facing cotton breeders is how to develop cotton varieties with diverse genetic backgrounds that are adapted to various adverse conditions. For example, facing a global scarcity of fresh water resources, drought has already become one of the major factors limiting cotton production, especially in Northwestern China cotton growing areas. Verticillium wilt (VW), a destructive disease that resulted into huge loss of the yield and quality of cotton in the last decade years, imperils sustainable cotton production in the future. However, numerous studies have shown that Upland cotton has a low level of genetic diversity, which it has been losing over the past century[1–7] due to the overuse of relatively few cultivars in larger areas for both breeding and production[7]. Thus, the existing genetic base of cotton should be broadened. *Gossypium arboreum* is a diploid cultivated cotton species (2n = 26, AA) native to Asia that possesses invaluable characteristics unavailable in the tetraploid cultivated cotton gene pool, such as resistances to pests (*Apolygus lucorum*) [8,9] and diseases (caused by *Verticillium dahliae*, *Fusarium oxysporum vasinfectum* and cotton leaf curl virus) [10–13] and tolerance to drought [14], which are extremely useful for the improvement of tetraploid (*G*. *hirsutum*) cotton.

However, when *G*. *hirsutum* as the maternal parent is pollinated with *G*. *arboreum* as the paternal parent, post-fertilization barriers further hinder or retard the development of the zygote after fertilization as well as normal seed development. To overcome incompatibility between *G*. *hirsutum* and *G*. *arboreum*, several researchers have attempted to develop embryo or ovule rescue techniques. For example, Beasley and Ting [15] found that a high salt BT medium (Beasley and Ting medium) supplemented with phytohormones made ovule grow well, but only two weeks of ovule and fiber development were reported. Stewart and Hsu [16,17] employed BTP (BT with phytohormone) medium supplemented with 10–15 mM ammonium ions to support ovule growth and embryo germination; the embryos grew very slowly, with reduced cotyledons, even after 10 weeks of culture in the absence of extra NH_4_^+^. Gill and Bajaj [18] employed ovule and embryo culture technique to develop complete plants from *G*. *arboreum* × *G*. *hirsutum* crosses and their reciprocals. They employed ovules at 3 d after pollination to culture them in a liquid medium for further profuse proliferation, whereas when the ovules were cultured on agar-solidified MS medium [19] supplemented with casein hydrolysate (CH), indoleacetic acid (IAA) and kinetin (Kin), they germinated and ultimately formed hybrid plants. Thengane et al. [20] excised 8–12 d ovules from cotton and cultured them on BT medium supplemented with IAA, gibberellic acid (GA_3_), ammonium chloride and CH. No single medium ensured complete development of the fertilized ovules to plantlets, thus necessitating a sequential five-step transfer process to different media, which ultimately led to the production of a *G*. *hirsutum* × *G*. *arboreum* hybrid. While Eid et al. [21] found that *G*. *hirsutum* ovules grew best on MS medium, Gill and Bajaj [18,22] successfully used MS medium supplemented with phytohormones and 300 mg l^-1^ CH to culture some *Gossypium* species. Sacks [23] determined that the medium composition significantly affects the germination of hybrid ovules of *G*. *hirsutum* × *G*. *arboreum*, with the highest frequency of ovule germination observed on MSB (MS with B5 vitamins [24]) supplemented with 1.9 g l^-1^ additional KNO_3_.

In addition to medium composition, previous studies on interspecific hybridization have shown that hybrid embryo development is strongly influenced by the paternal species used in the cross. Therefore, different cross combinations lead to various degrees of hybrid embryo development, which may be a more important factor than age or size at the time of embryo rescue. In general, immature embryos rescued prior to 15 days post anthesis (dpa) fail to show further development or undergo precocious germination. The optimum time for embryo rescue and ovule recovery occurs between 15 and 25 dpa [25].

In this study, to overcome the cross-incompatibility between these two parents for transferring favorable genes from *G*. *arboreum* into *G*. *hirsutum*, we improved an embryo rescue technique. Embryos prior to 3 dpa from the cross, *G*. *hirsutum* × *G*. *arboreum*, were put on MSBK supplemented with 250 mg l^-1^ CH and only one plant hormone, Kin to rescue. Then the putative interspecific hybrid F_1_ *G*. *hirsutum* × *G*. *arboreum* plants were produced and characterized by cytology, SSR markers and morphology. In addition, we treated the verified hybrids with different concentrations of colchicine in order to double the chromosome complements to generate amphiploids. And then, we confirmed the generation of amphiploids using molecular cytogenetic techniques. Finally, we preliminarily evaluated the resistances to Verticillium wilt (VW) and drought of S_1_ (derived from progenies of the new synthetic amphiploids self-pollinated) at seedling stage to explore the potential for cotton breeding.

## Materials and Methods

### Plant materials

The Asiatic diploid cultivated cotton *G*. *arboreum* cv Shixiya 1 (2n = 2x = 26, AA), a highly inbred line whose genome has been sequenced [26], possesses many desirable genes unavailable in tetraploid cotton, such as resistance to pests (*Apolygus lucorum*) [8,9] and diseases (infection by *Verticillium dahliae*, *Fusarium oxysporum vasinfectum* and cotton leaf curl virus) [10–13], tolerance to drought [14] and high fiber strength. Cultivated tetraploid cotton, *G*. *hirsutum* acc TM-1 (2n = 4x = 52, AADD), a genetic standard line, has been self-pollinated more than 60 times; its genome was also sequenced recently and will be released soon (personal communication).

*G*. *hirsutum* acc TM-1 and *G*. *arboreum* cv Shixiya 1 were grown in clay pots at Pailou experimental station, Nanjing Agricultural University, China (PES/NAU). *G*. *hirsutum* flowers were manually emasculated one day before anthesis and the pistils were covered with 4–6 cm straw tubes folded to prevent pollination. On the anthesis day, the emasculated flowers were pollinated with pollen from *G*. *arboreum*, and the pistils were again covered with folded straw tubes. The growth regulator GA_3_ (50 mg l^-1^) was then applied to the base of the pedicel of the cross-pollinated flowers to prevent shedding as per Qian et al. (1992) [27].

### Medium selection and ovule–embryo culture procedures

Young bolls were harvested at 3 days post anthesis (dpa), washed with soap and rinsed in water for 15–20 min. The surfaces of the bolls were then sterilized by soaking in ethanol (70%) for 1 min, followed by soaking in 3% NaHClO_3_ for 8–15 min in a laminar flow hood. The bolls were rinsed three times in sterilized distilled water, and the ovules were excised from the bolls using a scalpel. An average of 20 ovules was excised per boll. The ovules were cultured on three different types of media for 30 d to identify the most suitable medium for ovule growth, including MSB2K (MSB with twice the standard concentration of KNO_3_) [23], MSB2K supplemented with 1 mg l^-1^ IAA, 0.2 mg l^-1^ and 250 mg l^-1^ CH [18] and MSB2K supplemented with 0.5 mg l^-1^ Kin and 250 mg l^-1^ CH. The cultures were maintained under dark conditions at 26 ± 2°C for 7 to 9 weeks, and the expanded ovules were then transferred to MSB2K for germination. After an additional 20 days, young embryos germinated from the ovules. The germinated embryos were isolated and transferred to MSB basal medium. After the embryos developed into seedlings, they were transferred to MSB containing glucose (30 g l^-1^) instead of sucrose (30 g l^-1^). All of the germinated embryos were grown in a growth chamber under a 16 h/8 h light/dark and 26/24°C day/night temperature regime. Meanwhile, to ensure the each obtained seedling to survive, when they grew 6–8 cm tall, the seedlings were grafted to a stock of wilt-disease-resistant cotton, *Gossypium barbadense*.

### Characterization of the obtained putative hybrids

During flower development, the morphological traits of the putative hybrid plants were compared with those of their parents, including the following: stem color, stem hairiness, leaf color, leaf hairiness, leaf shape, leaf lobe, leaf texture, petiole color, petiole length, flower color, flower size, petal spots, petal length, petal width, stigma color, stigma length, anther color, anther number, bracteole dentations, bracteole length, bracteole width and pedicel length.

### DNA extraction, PCR amplification and gel electrophoresis

Young leaves from the two parents (*G*. *hirsutum* and *G*. *arboreum*) and the obtained hybrid plants (F_1_) were collected for extraction of total genomic DNA as per Paterson et al. [28]. Based on the high-density tetraploid cotton linkage map constructed in our laboratory [29], 1532 simple sequence repeat (SSR, microsatellite) primer pairs were randomly selected to screen for polymorphisms between the two parents and the putative hybrid plants. All SSR primer information for this work was from http://www.cottonmarker.org. Using a Peltier Thermal Cycler EDC-810 (Eastwin, Hong Kong), PCR amplification was performed in a volume of 10 μl containing 1 μl of DNA extract (20 ng μl^-1^), 1 μl of 5.0 μM of each primer, 1 μl of 2.50 mM MgCl_2_, 0.2 μl of 200 μM dNTPs, 0.1 μl of Taq polymerase (5 U μl^-1^), 1 μl of reaction buffer (10×) and 4.7 μl of ddH_2_O. The thermocycler conditions were as follows: one cycle of 95°C for 2 min, 30 cycles of 94°C for 45 s, 57°C for 45 s and 72°C for 60 s, and one cycle of 72°C for 7 min. PCR products were determined by electrophoresis as described by Zhang et al. (2000, 2002)[30,31].

### Chromosome observation

Root tips were used to observe mitotic metaphase, and young buds approximately 3–4 mm long were used for meiotic metaphase chromosome preparations.

Young flower buds of the hybrids were collected between 8:30 to 10:00 a.m. Meiotic chromosome spreads were prepared as per Wang et al. [32] with several modifications. The collected buds were fixed in ethanol–acetic acid (3:1) fixative for 2–24 h at 4°C after removing the calyx and corolla. Then, buds containing pollen mother cells (PMC) in metaphase I were selected, from which several anthers were placed onto ethanol-washed glass slides with a drop of 45% acetic acid (v/v), freed of debris and squashed. The slides were stained in 4’6-diamidino-2-phenylindole (DAPI; Roche Diagnostics) for 10 min at room temperature, and anti-fade (Vector, USA) was applied under the coverslips. After that, the slides were examined under an Olympus BX51 fluorescence microscope. Chromosome images were captured using an evolution VF CCD camera (Media Cybernetics, Bethesda, MD, USA), and image processing was performed using Image-Pro express software (Media Cybernetics, Bethesda, MD, USA).

Mitotic chromosome preparation was carried out as per Wang et al. [32] with some modifications. To enrich metaphase cells, root tips were incubated in 25 μg/ml cycloheximide at 29°C for 2 h before they were excised from the S_1_ germinated seeds (self-pollinated from the hexaploid plants). After that, the root tips were collected and fixed in a solution of ethanol: acetic acid (3:1 v/v; Carnoy's Fluid) for an additional 24 h. After fixation, the root tips were macerated in 4% cellulase and 1% pectinase at 37°C for 40 min and fixed in Carnoy's Fluid for more than 2 h; the treated root tips were stored in 70% ethanol at -20°C for later observation on chromosome. The treated root tips in a drop of 45% acetic acid were squashed onto slides to disperse the cells and to allow the metaphase chromosomes to spread out. Slides with good metaphase spreads were stored at -70°C more than 12 h until use for genomic in situ hybridization (GISH) analysis as per Chen et al. [33].

### Colchicine treatment

In 2013, the interspecific hybrid plants were preserved and propagated by grafting. The stem apices of vegetative branches from these hybrid plants were immersed in 0.05, 0.10, 0.15 and 0.20% (w/v) colchicine solution for 24, 36 and 48 h and grown in ceramic pots under natural conditions. Each treatment employed 30 stem apices.

### Preliminary assessment of drought tolerance for S_1_ derived from progenies of new synthetic amphiploids self-pollinated

These two parents of amphiploids, TM-1 and Shixiya 1, were used as control to assess S_1_ tolerance to drought. Seeds were delinted with 98% H_2_SO_4_ and then soaked in 70% ethanol for 5 min and in 10% H_2_O_2_ for 2 h to sterilize the surface, followed by three rinses with sterile water. Surface-sterilized seeds were germinated at 26°C light incubator on plates containing sterilized water for 24h and then the seedlings were grown on 1/2 MS medium for 2 d. Fifteen uniform seedlings for each in a randomized block design with three replicates were grown in paper cups of 7.3 × 5.1 × 8.3 cm with an autoclaved mixture of vermiculite and peat (1:1 v/v) in the greenhouse at PES/NAU. When cotton grew to three-leaf stage, drought-stress treatments were performed. For drought treatment, plants were not watered for 8 d to create drought conditions, while for control, plants were watered regularly to grow. The plants were monitored daily for wilting. The number of wilted leaves and wilted plants, plant height, shoot and root dry weights were recorded. Relative water content (RWC) was calculated to assess drought tolerance.

Three physiological parameters, i.e., photosynthesis rate, stomatal conductance, transpiration rate, were also employed to evaluate drought tolerance. After 5 days of drought treatment, the photosynthesis rate, stomatal conductance and transpiration rate were determined by a portable photosynthesis system (Li-6400XT, LICOR Inc., Lincoln, NE, USA). As per the manufacturer’s guidelines, the instrument was stabilized for measurement. Under the conditions of a CO_2_ concentration of 400 μmol mol^-1^, a relative humidity of 50%, a chamber temperature of 28°C, and a photon flux density of 1200 μmol m^-2^s^-1^, the photosynthesis rate, stomatal conductance and transpiration rate were measured. All the measurements were performed from 9:00 to 10:00 a.m.

### Determination of relative water content of leaves

The first fully expanded leaves from well-watered as well as water-stressed plants were collected to determine their fresh weights (FW). After that, each leaf was soaked in water in a Petri dish, sealed with parafilm to absorb water for 24 h at room temperature. After full absorption of water, leaves were blotted on filter paper to remove excess water and determined their turgid weights (TW). Dry weights (DW) were weighed after drying the leaves in an oven for 72 h at 70°C. Relative water content (RWC) was calculated according to the following formula as described by Schonfeld et al. [34]:

RWC(%)=(FW−DW)/(TW−DW)×100.

### Preliminary assessment of Verticillium wilt resistance for S_1_ derived from progenies of amphiploids self-pollinated

Both parents and S_1_ were evaluated for resistance to Verticillium wilt (VW). *G hirsutum* cv. Junmian 1, distributed widely in Xinjiang Uygur Autonomous Region and highly sensitive to VW, was employed as the control. One defoliating *V*. *dahliae* isolate V991 [35,36], with extra-strong virulence, discovered and isolated by the Institute of Plant Protection, Jiangsu Academy of Agricultural Sciences in Changshu City, Jiangsu Province of China in 1991, are widely used for evaluation of cotton resistance to VW now. Here, the isolate V991 was selected to represent isolate for evaluation of cotton resistance to VW. The *V*. *dahliae* isolate was grown on potato dextrose agar plates at 25°C for 10–14 d. Inocula for experiments were prepared by spreading a conidial suspension no agar plates that were then incubated at 25°C for 6–7 d. Conidia were then collected and diluted to 1 × 10 ^7^ cells mL^-1^ for inoculation.

The uniform seedlings were grown in paper cups of 7.3 × 5.1 × 8.3 cm with an autoclaved mixture of vermiculite and peat (1:1 v/v) in the greenhouse at PES/NAU. These seedlings were planted in a randomized block design with three replicates. *V*. *dahliae* V991 were used to inoculate seedlings individuals (after the emergence of two true leaves) by watering with 15 mL of conidial suspension. Disease symptoms were scored every three days after 11 day post inoculation (dpi), according to a national standard for screening cotton for VW resistance in China [37,38]: grade 0, healthy with no disease symptoms; 1. < 25% chlorotic/necrotic leaves; 2. 25~50% chlorotic/necrotic leaves; 3. 50~75% chlorotic/necrotic leaves; 4. > 75% chlorotic/necrotic leaves or killed by disease. Single plant numbers and grades were recorded at every time point, and the disease index (DI) and relative DI (RDI) of the tested canopy were calculated according to the following formulae:

$$\begin{array}{l} DI\left(\%\right)=\left[\;\sum \left(f-i\right)/\left(f\times 4\right)\;\right]\times 100\\ RDI\left(\%\right)=K\times DI\\ K=50\%/DI\;\text{of control} \end{array}$$

Where “i” denotes the grade of disease severity, and “f” is the number of plants in each grade. The RDI values were used to divide disease severity of the test canopies to VW into five grades: immunity, RDI = 0; high resistance (HR), RDI < 10.0%; resistance (R), RDI (%) = 10.1–20.0; tolerance (T), RDI (%) = 20.1–35.0; and susceptibility (S), RDI > 35.0%.

### Statistical analysis

All data were presented as mean ± standard deviation (SD) at three independent replicates, and comparisons among two parents, S_1_ and the controls were performed using one way ANOVA with Duncan’s multiple range test. A value of P < 0.05 was considered to be statistically significant. All statistical analyses were done using IBM SPSS Statistics (IBM Corporation, New York, USA).

## Results

### Production of interspecific hybrids between *G*. *hirsutum* and *G*. *arboreum*

**Fig 1:**
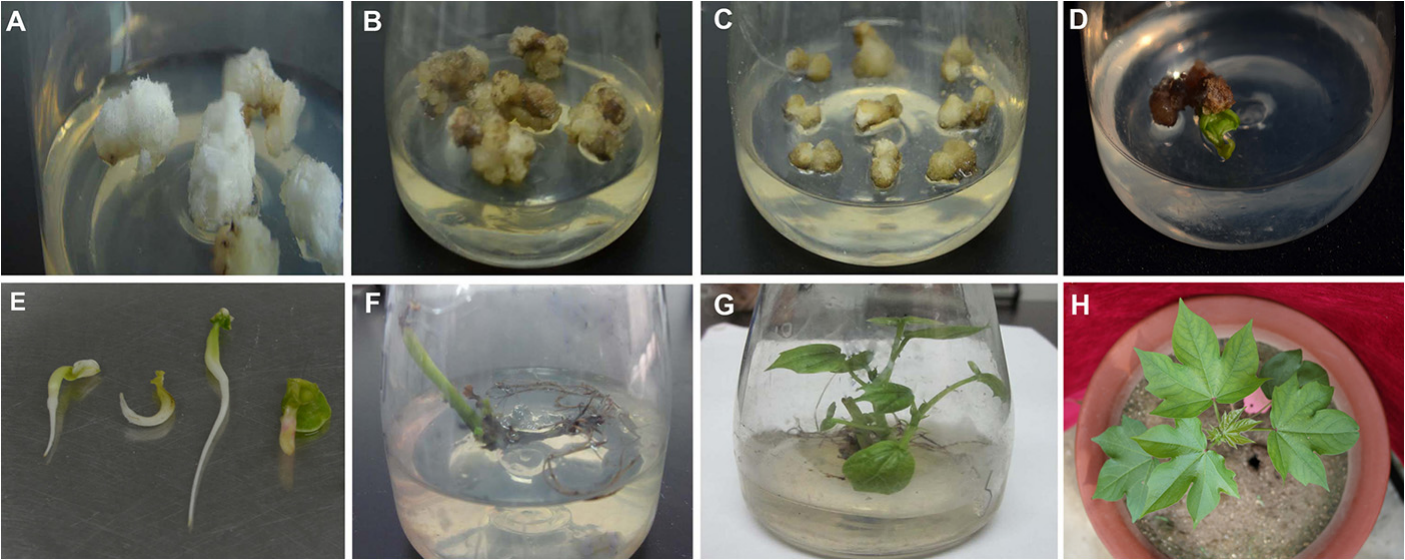
Embryo rescue of *G*. *hirsutum* × *G*. *arboreum* hybrid ovules. A, Ovules on MSB2K produced numerous fibers and calli; B, Ovules on MSB2K+1.0 mg l^-1^ IAA+0.2 mg l^-1^ Kin+250 mg l^-1^ CH produced numerous calli; C, Ovules on MSB2K+0.5 mg l^-1^ KIN+250 mg l^-1^ CH grew well, with little callus production; D, Ovule germination; E, Embryo isolation; F, All leaves of plantlets dropped; G, Healthy plantlets; H, Grafted plantlets.

Medium composition significantly affects the growth and germination of ovules [23], with different genotypes responding to different media [25]. Sacks [23] used MSB2K medium without growth regulators and obtained efficient ovule growth for *G*. *hirsutum* × *G*. *arboreum*. However, in the current study, only fibers and calli grew well on MSB2K medium, while ovules did not (**Fig 1A**), which revealed that the ovules did not grow well on medium lacking growth regulators. Gill and Bajaj [18] and Thengane et al. [20] also failed to germinate *G*. *hirsutum* × *G*. *arboreum* ovules on medium lacking growth regulators. These results are inconsistent with the results of Stewart and Hsu [17], who found that more *G*. *hirsutum* × *G*. *arboreum* hybrid seedlings were produced on medium without growth regulators than on media containing various combinations of IAA and Kin. We also found that more calli emerged on the medium used by Gill and Bajaj [18] than on medium containing only one plant hormone, i.e., Kin (**Fig 1B**). Thengane et al. [20]found that cultured ovules produce more callus on medium containing IAA than on medium without this hormone. Therefore, we improved the culture medium used for embryo rescue by removing the IAA and increasing the concentration of Kin. The results indicate that embryos developed well on this medium, while they did not develop fibers, and little callus was produced (**Fig 1C; Table 1**). The embryos were cultured on MSB2K medium supplemented with 0.5 mg l^-1^ Kin and 250 mg l^-1^CH as basal medium for 30 days (**Table 2**). The results showed that ~ 97% of the ovules were viable and 91% of the embryos expanded (**Table 3**). Therefore, we believe that MSB2K medium containing 0.5 mg l^-1^ Kin and 250 mg l^-1^ CH is suitable medium for hybrid embryo rescue.

**Table 1:** Media used in the first 30 days in culture (dac) for 3 dpa embryo rescue (100 ovules cultured on medium).

Medium	Ovule’s viability (%)	Ovule growth
MSB2K	94	+ +
MSB2K+1.0 mg l^-1^ IAA+0.2 mg l^-1^Kin+250 mg l^-1^ CH	96	+
MSB2K+0.5 mg l^-1^ Kin+250 mg l^-1^ CH	97	+++

+++, little callus

++, callus with more fibers

+, more callus

**Table 2:** Culture procedures and media used in this study.

Embryo developmental stage	Culture time (dac)	Medium composition
Ovule enlargement	0–65	MSB2K+0.5 mg l^-1^ Kin + 250 mg l^-1^ CH
Ovule germination	65~90	MSB2K
Embryo develops into a seedling	90~100	MSB+ 1.0 mg l^-1^ Kin
Root formation	100~102	MSB+0.5 mg l^-1^ NAA+1.0 g l^-1^AC
Healthy plantlets	100~120	MSB+30 g l^-1^ Glucose

**Table 3:** Ovules cultured, percentage of viable ovules at 20 d after culture and embryo emergence, number of embryos germinated and number of F_1_ plants obtained.

No. of bolls used for ovule collection	No. of ovules cultured	Ovule viability (%)	Rate of embryo expanded (%)	No. of germinated embryos	No. of plants
19	276	97	91	10	8

After an additional 20–35 d of culture on this medium in the dark, we transferred the ovules to MSB2K without plant hormones for ovule germination. The ovules were continuously cultured on the same medium for 20 d in the light. Under these conditions, ten ovules developed into embryos and were isolated (Fig 1D and 1E), followed by culture for 20 d on MSB with 1.0 mg l^-1^ Kin for cotyledon growth. After a total of approximately 100 d of culture, nine embryos developed into seedlings, while one died. We transferred the eight seedlings to MSB containing 0.5 mg l^-1^ NAA and 1.0 g l^-1^ activated carbon (AC) for root formation. The plantlets formed large roots after approximately 2 d of culture on this medium. After the plantlets developed 3–4 leaves, the leaves began to fall off from the plantlets, which may have been caused by the high ion or hormone concentration in the medium. Therefore, we used 1/2 MS medium containing half of the normal sucrose concentration and no plant hormones. However, leaves continued to drop off from the plantlets, with no leaves remaining after one week (**Fig 1F**). Finally, the seedlings were transferred to MSB medium containing glucose (30 g l^-1^) in place of sucrose. The plantlets grew well on this medium **(Fig 1G**), perhaps because glucose produces lower osmotic pressure than sucrose, or perhaps because glucose is easier for weak seedlings to absorb. When the seedlings were 6–8 cm tall, they were grafted onto a stock of *G*. *barbadense* for further growth (**Fig 1H**).

### Morphological characteristics of the obtained putative F_1_ hybrids

**Fig 2:**
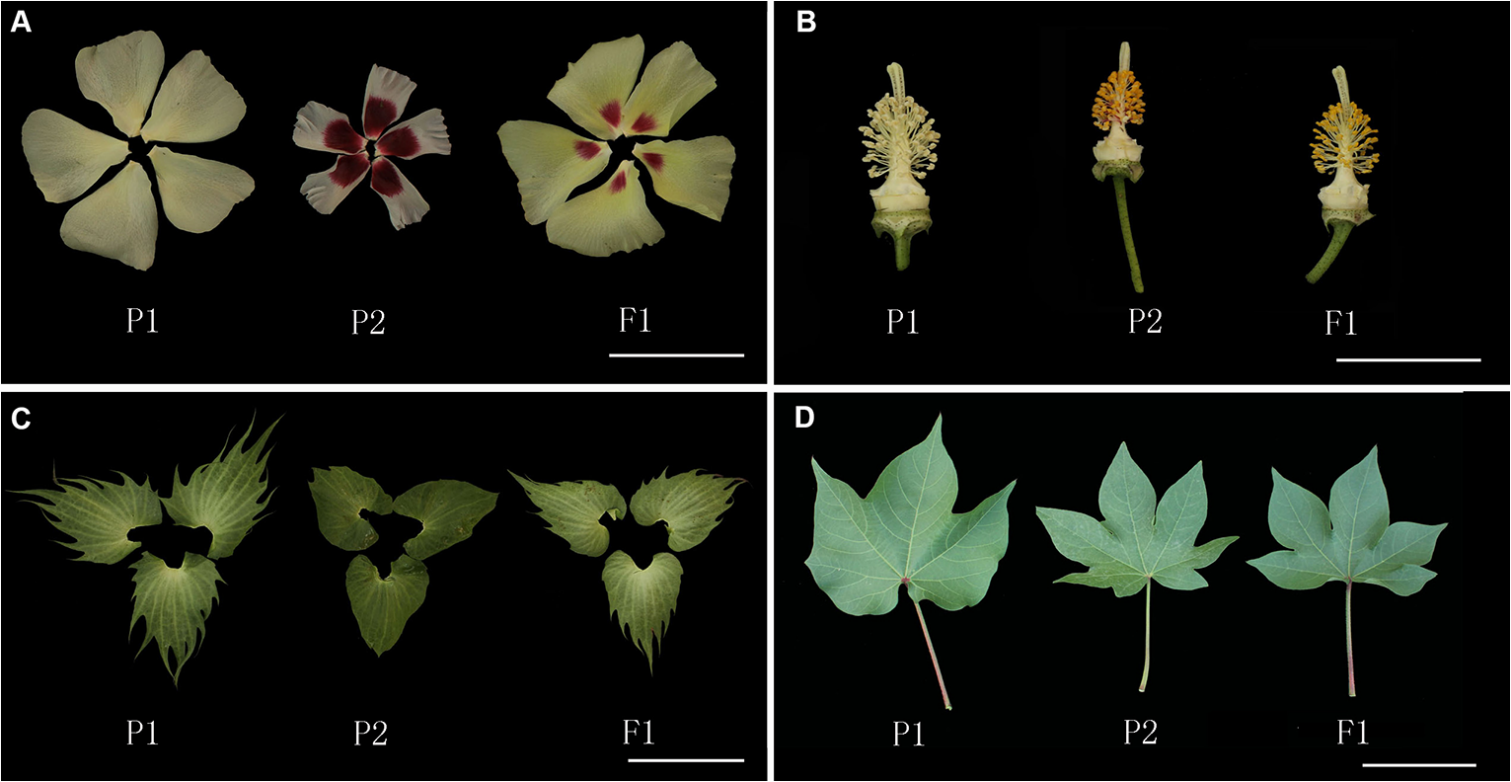
Morphology of *G*. *hirsutum*, *G*. *arboreum* and F_1_ hybrids. A, Petals; B, Pistils and stamens; C, Bracts; D, Leaves. Scale bars, 50 mm

All of the obtained putative F_1_ hybrids were highly similar in terms of morphology. We examined 22 morphological traits of the putative hybrids during flowering, including the color, shape and size of stems, leaves, flowers and so on **(Table 4)**. The result indicate that most traits tended to resemble those of the paternal parent, *G*. *arboreum*, e.g., light red stem, pubescent stem, light green leaf, slightly hairy leaf, deeply lobed leaf, leaf lobe, leaf texture, petiole color and petiole length. A few traits resembled those of the maternal parent, *G*. *hirsutum*, e.g., petal length and width, stigma length, anther number and pedicel length. Some traits showed intermediate phenotypes, e.g., petal color, petal spot and anther color. These putative hybrids were highly sterile when crossed with TM-1 regardless of whether they were used as the male or female parent (**Fig 2**).

**Table 4:** Morphological characteristics of the obtained putative F_1_ hybrids.

Characters	*G*. *hirsutum*	*G*. *arboreum*	*G*. *hirsutum* × *G*. *arboreum* (3x)	*G*. *hirsutum × G*. *arboreum* (6x)
Stem color	Green	Light red	Light red	Light red
Stem hairiness	Light pubescent	Pubescent	Pubescent	Pubescent
Leaf color	Green	Light green	Light green	Deep green
Leaf hairiness	Hairy	Slight hairy	Slight hairy	Slight hairy
Leaf shape	Shallow lobed	Deeply lobed	Deeply lobed	Deeply lobed
Leaf lobe number	2–4	2–6	2–6	2–6
Leaf texture	Thin soft	Thick hard	Thick hard	Thick hard
Leaf length (cm)	11.32±1.16	8.29±0.79	7.22±0.70	15.82±1.36
Leaf width (cm)	14.48±1.65	9.80±0.89	8.38±0.90	16.47±1.43
Petiole color	Green	Light green	Light green	Deep green
Petiole length (cm)	9.97±1.36	7.18±0.60	10.68±0.52	11.24±1.32
Petal color	Creamy	White	Yellow	Yellow
Flower size	Large	Small	Medium	Large
Petal spot	Absent	Big dark red	Medium dark red	Big dark red
Petal length (cm)	4.85±0.27	2.91±0.23	4.35±0.23	5.29±0.19
Petal width (cm)	4.98±0.27	3.16±0.20	4.77±0.46	5.79±0.70
Stigma color	Creamy	Creamy	Creamy	Creamy
Stigma length (cm)	1.00±0.13	0.65±0.11	0.90±0.17	1.1±0.12
Anther color	creamy	Yellow	Light yellow	Yellow
Anther number	121.50±4.67	110.40±3.44	120.70±2.58	112.60±5.06
Fertility	Fertile	Fertile	Sterile	Fertile
Bracteole dentation shape	Very long	Very short	Long	Long
Bracteole dentation number	10–13	4–6	10–13	10–13
Bracteole length (cm)	5.04±0.39	3.24±0.60	4.28±0.55	5.76±0.12
Bracteole width (cm)	3.85±0.41	2.23±0.18	3.45±0.41	4.71±0.64
Pedicel length (cm)	1.7±0.36	3.24±0.60	1.7±0.24	2.97±0.49

### Cytological observation of the chromosome configurations of putative hybrid F_1_ plants at metaphase I during meiosis

**Fig 3:**
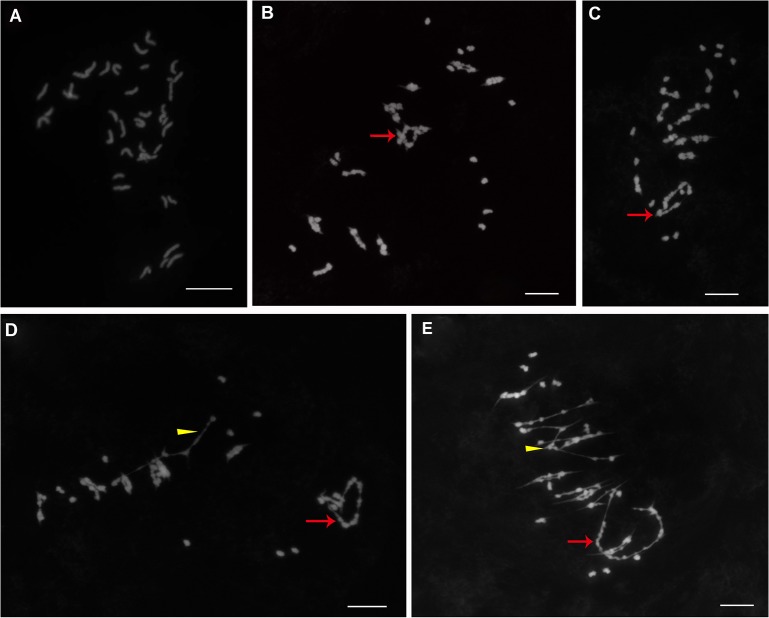
The putative interspecific F1 hybrid was confirmed by cytogenetic observation on mitotic cell and chromosome configuration at metaphase I of meiosis in pollen mother cells (2n = 3x = 39). A: Mitotic cell of interspecific F_**1**_ hybrid. B and C: 13I+10II+1VI. D and E: 13I+8II+1IV+1VI. Yellow arrowheads indicate quadrivalents and red arrows indicate hexavalents. Scale bars, 10 μm.

Cytological data for the obtained putative hybrids of *G*. *hirsutum* × *G*. *arboreum* are summarized in **Table 5**. All of the cells observed had 39 chromosomes, indicating that they were triploid (2n = 3x = 39; **Fig 3A**), which confirmed that the putative hybrids were derived from interspecific hybridization between *G*. *hirsutum* and *G*. *arboreum*. The results also demonstrate that the chromosome configurations in the hybrids were variable, with uni-, bi-, tri-, quadri-, penta-, hexa- and octavalents. Of the 81 pollen mother cells observed, most cells (18/81) showed 13 univalents, eight bivalents, one quadrivalents and one hexavalent, followed by cells (9/81) showing nine univalents, 10 bivalents, one quadrivalent and one hexavalent. The average chromosome configurations were 13.38 uni-, 8.25 bi-, 0.15 tri-, 0.95 quadri-, 0.05 penta-, 0.69 hexa- and 0.06 octavalents. The number of univalents ranged from five to 31, with 13 being the most frequent number, followed by 11 and 15. The number of bivalents ranged from four to 12, with eight being the most frequent number followed by 10 and nine. The most highly represented multivalents were quadrivalents, with an average of 0.95, followed by hexavalents, with an average of 0.69, which indicates that two translocation events occurred involving two and three pairs of chromosomes between these two species, respectively, which is consistent with the observations of Gerstel and Sarvella [39] (**Fig 3B–3E**). The high frequency of univalents in pollen mother cells (PMC) at metaphase I in meiosis explained why the putative F_1_ hybrids were highly sterile, since due to disordered segregation, univalents were often lost at Anaphase I, leading to the formation of imbalanced, non-viable gametes lacking a complete set of chromosomes.

**Table 5:** Chromosome configurations of PMC at metaphase I of meiosis.

No. of PMCs	I	II	III	IV	V	VI	VIII	No. of chromosomes
1	31	4						39
1	29	5						39
1	25	4				1		39
1	24	4	1	1				39
2	21	9						39
1	19	10						39
1	18	4	1	1		1		39
1	18	5	1	2				39
1	18	6	1			1		39
1	17	9		1				39
2	17	8				1		39
1	15	9				1		39
2	15	8		2				39
1	15	10		1				39
1	15	9				1		39
1	15	10		1				39
1	15	8		2				39
1	15	7		1		1		39
1	15	5		2		1		39
1	14	7			1	1		39
1	13	4	2	1			1	39
1	13	10	2					39
2	13	8		1		1		39
1	13	9					1	39
1	13	4		3		1		39
18	13	8		1		1		39
3	13	9		2				39
1	13	5		1		2		39
1	12	9	1			1		39
1	12	7			1		1	39
1	12	7	1	1		1		39
1	12	10	1	1				39
1	11	10	1		1			39
1	11	7		2		1		39
2	11	9		1		1		39
1	11	12		1				39
2	11	9		1		1		39
1	11	11				1		39
1	11	8		1			1	39
1	11	7		2		1		39
1	10	10		1	1			39
1	9	11		2				39
2	9	8		2		1		39
9	9	10		1		1		39
1	9	11					1	39
1	7	9		2		1		39
1	5	12		1		1		39
Range	5~31	4~12	0~2	0~2	0~1	0~2	0~1	39
Average	13.38	8.25	0.15	0.95	0.05	0.69	0.06	39

### Validation of the obtained putative hybrids using SSR markers

**Fig 4:**
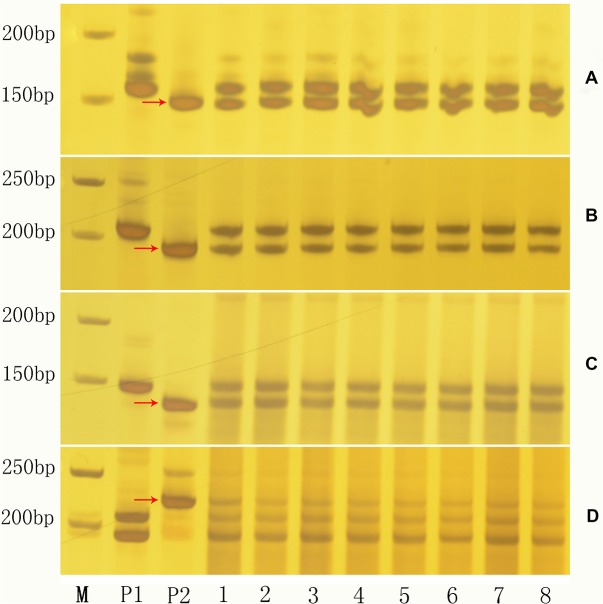
Validation of putative hybrids of *G*. *hirsutum* × *G*. *arboreum* using a randomly selected set of *G*. *arboreum*-specific SSR primers. From A to D, *G*. *arboreum*-specific amplicons (arrows) were detected using individual chromosome-specific SSR primers NAU70, NAU299, NAU740 and NAU815, respectively. P1, *G*. *hirsutum*; P2, *G*. *arboreum*; 1–8 show F_**1**_ plants of *G*. *hirsutum* × *G*. *arboreum*; M, molecular marker sizes (50 bp ladder).

We used a total of 1,532 SSR primer pairs/combinations selected from the linkage maps of the *G*. *hirsutum* and *G*. *barbadense* genomes constructed at our institute [29] to screen polymorphic primers between *G*. *hirsutum* and *G*. *arboreum*. The results indicate that approximately 82.0% (1,256/1,532) of the SSRs showed polymorphism between these two species. Of the 1,256 pairs of polymorphic primers used to characterize the putative interspecific hybrids, 513 (40.8%) showed codominance in the putative hybrids, whereas 687 (54.7%) were dominant to *G*. *hirsutum* and 56 (4.5%) were dominant to *G*. *arboreum*. the amplicons generated by codominant primers in the putative hybrids demonstrated that the hybrids had DNA bands from both parents, confirming that the putative hybrids were derived from *G*. *hirsutum* and *G*. *arboreum* (**Fig 4**).

### Generation of the amphiploid obtained by doubling chromosome complements in interspecific F_1_ hybrids between *G*. *hirsutum* and *G*. *arboreum*

**Fig 5:**
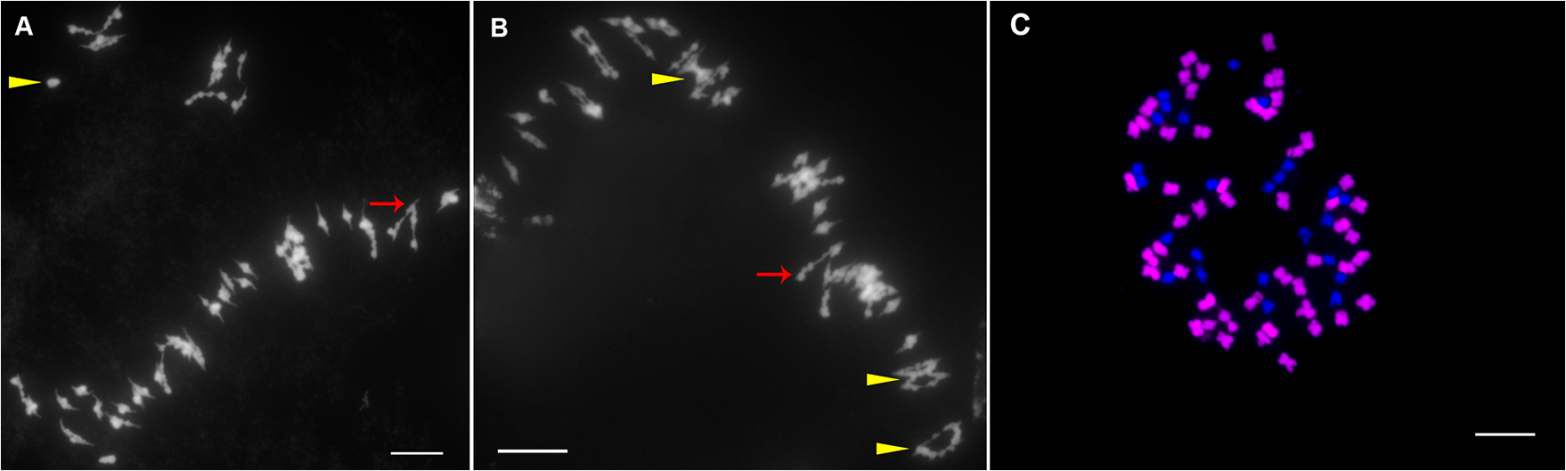
The amphiploids obtained by doubling chromosome complements in interspecific F_1_ hybrids between *G*. *hirsutum* and *G*. *arboreum* were confirmed by observation on chromosome configuration at metaphase I of meiosis in pollen mother cells and GISH on somatic cells. A, Univalent, bivalents and trivalent. Arrowhead (yellow) and arrow (red) indicate the univalent and trivalent, respectively. B, Bivalents, quadrivalents and pentavalent. Arrowhead (yellow) and arrow (red) indicate the quadrivalent and pentavalent, respectively. C, Red signals indicate the 52 chromosomes of A subgenome of *G*. *hirsutum* and *G*. *arboreum* and blue signals indicate the 26 chromosomes of D subgenome of *G*. *hirsutum* (stained with DAPI). The results demonstrate that the amphiploid is hexaploid and its genome component is 2n = 6x = AAAADD = 78. Scale bars, 10 μm.

The putative hybrid F_1_ plants, which were confirmed by cytology, molecular markers and morphological observation, were treated with different concentrations of colchicine for 24, 36 and 48 h. The results show that among 30 plants treated with 0.1% colchicine for 24 h, 22.22% (four out of 18 plants survived, while 12 died) of interspecific hybrid F_1_ plants exhibited gigantism (**Table 4**) and had improved fertility, and they set bolls and produced several seeds. To understand why these plants produced seeds, we collected young flower buds from these plants and observed their chromosome associations. The results indicate that the number of univalents significantly decreased while the numbers of bivalents and multivalents greatly increased (Fig 5A and 5B), even if there were too many chromosomes to discriminate, which suggests that these plants were chromosome-doubled and were therefore putative amphiploids, i.e., hexaploids. Therefore, to further verify the genome components of the putative amphiploids, S_1_ plants derived from the putative amphiploids by self-pollination were used to identify chromosome numbers by GISH using somatic mitotic cells of the root tips. The results demonstrate that their genome components contained four sets of A genome (red signals) and one set of D subgenome (blue signals) and had a total of 78 chromosomes, i.e., 2n = 6x = AAAADD = 78 (**Fig 5C**), which further confirms the authenticity of the amphiploids. Now S_1_ plants grow very well.

These data also show that the interspecific hybrid F_1_ plants were chromosome-doubled only after 0.1% colchicine treatment for 24 h, while no other hybrid F_1_ plants were found to be chromosome-doubled after treatment with other concentrations of colchicine for different periods of time, which implies that treatment with 0.1% colchicine for 24 h is suitable for cotton chromosome doubling.

### Preliminary assessment of drought tolerance for S_1_ derived from progenies of new synthetic amphiploids self-pollinated

Facing a global scarcity of fresh water resources, drought has already become one of the major factors limiting cotton production, especially in Northwestern China cotton growing areas. Improvement of cotton drought tolerance is the most economic and effective way of alleviating scarcity of water resources. *G*. *arboreum* cv Shixiya 1, a highly inbred line, is highly tolerant to drought [14] and *G*. *hirsutum* acc. TM-1, is sensitive to drought. Here, we evaluate the drought tolerance of S_1_ to explore the possibility of transference from *G*. *arboreum* into Upland cotton by the combination of morphological traits, leaf relative water contents and physiological traits, which will lay the foundation for mining and transference of drought resistance genes from *G*. *arboreum* into Upland cotton.

### Morphological traits were measured during drought stress

#### Wilting of leaves and plants

**Fig 6:**
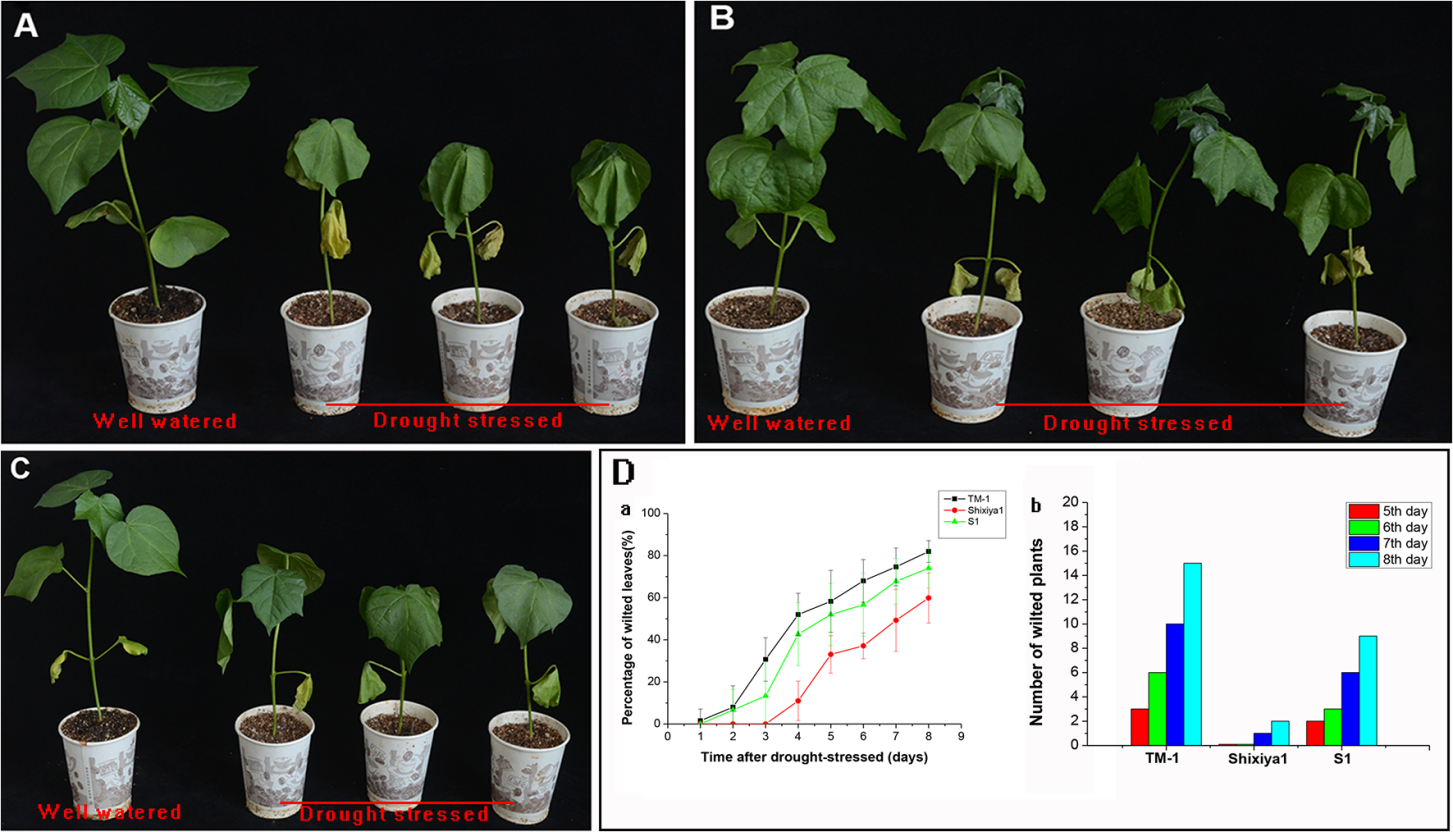
Phenotypes of 8-day drought treatment and percentage of leaves wilted and wilted plants after drought stress. A, TM-1; B, Shixiya 1; C, S_**1**_; D-a, Percentage of wilted leaves after drought-stressed; D-b, Number of wilted plants after drought-stressed.

The two parents as controls and S_1_ plants grown in paper cups were subjected to drought stress in a greenhouse by withholding watering for 8 days (Fig 6A–6C). At 8 days after drought treatment, leaves of 81.9% for TM-1 showed wilted, followed by 74.0% for S_1_ and 59.9% for Shixiya1, respectively (Fig 6D-a). During the whole drought treatment, TM-1 showed the most number of wilted plants (≥ 75% of leaves wilted) and the severest wilted while Shixiya is the least and the lightest. The wilting degree of S_1_ displayed was between these two parents (Fig 6D-b).

#### Relative water content of leaves

After 8 days of withholding of watering, RWC of two parents and their amphiploids decreased significantly (**Table 6**). However, Shixiya 1 had the highest leaf RWC with 70.09%, followed by S_1_ with 61.35%, being significantly higher (P<0.001 and P<0.05, respectively) than that of TM-1 with 56.49%. From leaf RWC reduction, the results also demonstrated that Shixiya 1 was the least amount of water lost during drought-stressed treatment while TM-1 was the highest and S_1_ was between two parents.

**Table 6:** Leaf relative water contents were measured with well-watered and 8-day drought-stressed.

Materials	Leaf relative water content (%)		Reduction (%)
	Watered	Drought	
TM-1	80.86±1.68	56.49±1.00	30.14**
Shixiya1	82.50±1.53	70.09±3.06**	15.04**
S_1_	81.19±2.61	61.35±5.51*	24.43**

* and ** indicate statistical significance at the 0.05 and 0.01 probability level, respectively.

#### Growth performance of cotton seedlings

After the treatment of 8-day drought stress, all plant grew slowly and their heights decreased than well-watered. Shixiya 1 showed the highest and reduced only by 18.25% and TM-1 showed the most dwarf and decreased by as low as 34.94%, while the S_1_ plants showed between its parents and decreased by 27.9%. Shoot dry weight also showed the similar results. Shixiya 1 plants showed the highest shoot dry weight with 686.11 mg/plant and reduced by 20.11% and TM-1 showed the lightest with 611.85 mg/plant and decreased by 30.02%, while the S_1_ plants also showed between its parents. For root dry weight, Shixiya 1 plants less increased by 8.31% and S1 by up to 11.51% followed by TM-1 by 10.15% (**Table 7**).

**Table 7:** Growth performance of cotton seedlings with well-watered and 8-day drought-stressed.

Material	Plant height (cm)			Shoot dry weight (mg/plant)			Root dry weight (mg/plant)		
	Watered	Drought	Reduction (%)	Watered	Drought	Reduction (%)	Watered	Drought	Increase (%)
TM-1	10.40±0.49	6.77±0.59	34.94**	874.38±57.86	611.85±34.70	30.02**	343.27±22.18	368.12±32.95**	10.15**
Shixiya1	13.92±0.40	11.38±0.49**	18.25**	858.82±39.37	686.11±29.36*	20.11**	256.86±19.36	278.20±10.61	8.31**
S_1_	10.71±0.97	7.72±0.70*	27.90**	899.89±52.69	679.75±28.70*	24.46**	361.26±17.28	402.84±39.11**	11.51**

* and ** indicate statistical significance at the 0.05 and 0.01 probability level, respectively.

#### Photosynthetic performance under drought stress

To understand the physiological mechanisms of drought tolerance, net photosynthesis rate, stomatal conductance and transpiration rate of leaves were measured during the period of drought stress at 5^th^ day of plants of all lines. On the well watered conditions, net photosynthesis rate and stomatal conductance of TM-1 are significantly higher than those of Shixiya 1 and S_1_. But under drought stress conditions, net photosynthesis rates, stomatal conductance of TM-1 decreased rapidly and even lower than those of Shixiya1 and S_1_. Whether under treatment or not, transpiration rate of TM-1 was always lower than those of Shixiya1 and S_1_ (**Table 8**). The results indicated that under drought stress, net photosynthesis rate, stomatal conductance and transpiration rate of leaves in Shixiya 1 and S_1_ remained relatively higher than those in TM-1, implying Shixiya 1 and S_1_ possessed better drought tolerance than TM-1.

**Table 8:** Effects of drought stress on photosynthesis, stomatal conductance and transpiration rate.

Material	Net photosynthesis rate (μmol CO_2_ m^-2^s^-1^)		Stomatal conductance (mmol H_2_O m^-2^s^-1^)		Transpiration rate (mmol H_2_O m^-2^s^-1^)		
		
	Watered	Drought	Watered	Drought	Watered	Drought	
TM-1	6.19±1.10	0.56±0.18	39.53±9.70	4.35±0.85	1.10±0.25		0.14±0.05
Shixiya1	5.58±0.23	0.99±0.26*	34.28±3.95	6.04±1.26*	1.14±0.15		0.21±0.05*
S_1_	5.61±0.97	0.76±0.28*	38.81±6.40	5.30±3.48	1.23±0.37		0.17±0.09

* indicates statistical significance at the 0.05 probability level.

Based on the results of wilting of leaves and plants, relative water content of leaves, growth performance of cotton seedlings and Photosynthetic performance, Shixiya 1 is highly tolerant to drought and the tolerance can be transferred into Upland cotton by hybridization.

### Preliminary assessment of Verticillium wilt resistance for S_1_ derived from progenies of amphiploids self-pollinated

**Fig 7:**
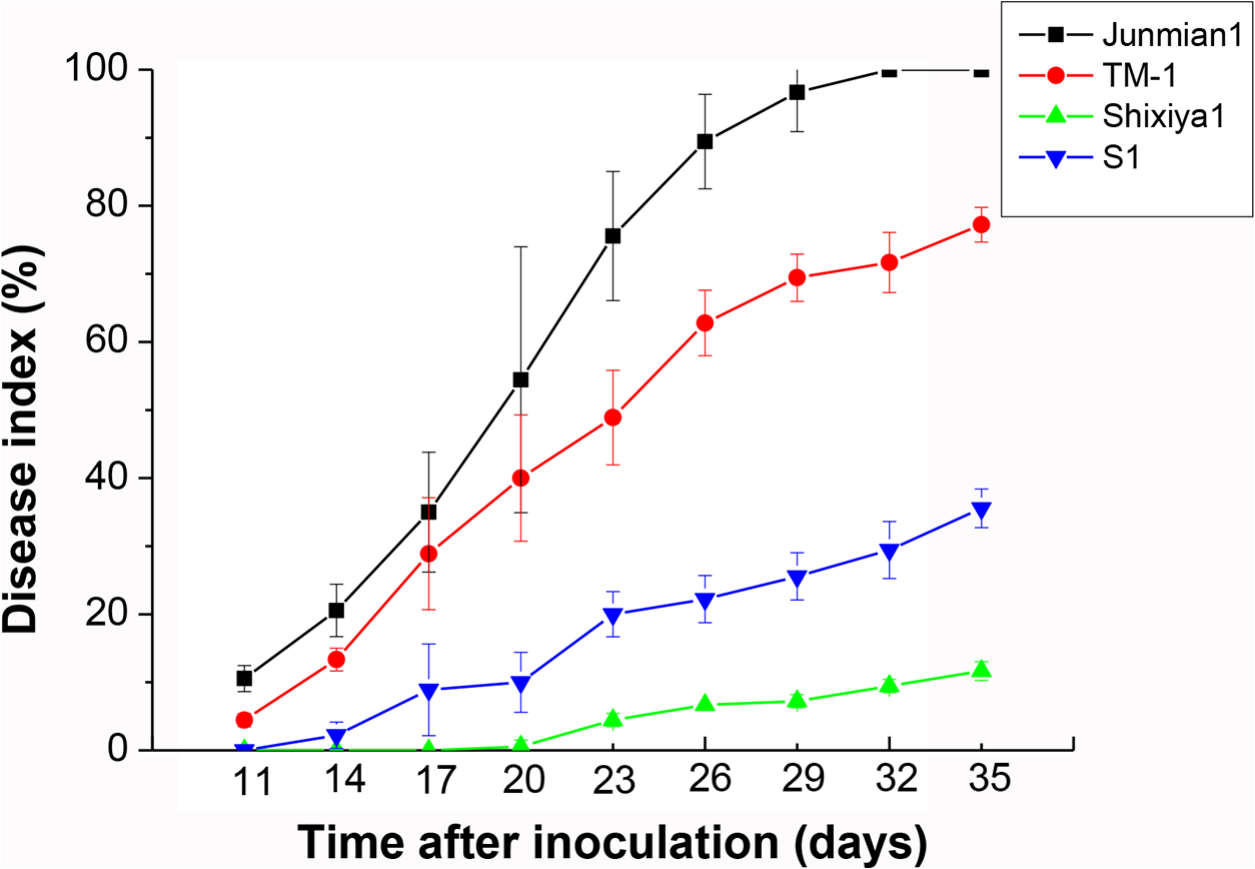
Rate of disease index during 35 days infected by *Verticillium dahliae* isolate V991. The experiments were repeated in three replicates. The error bars were calculated based on three biological replicates using standard deviation.

**Fig 8:**
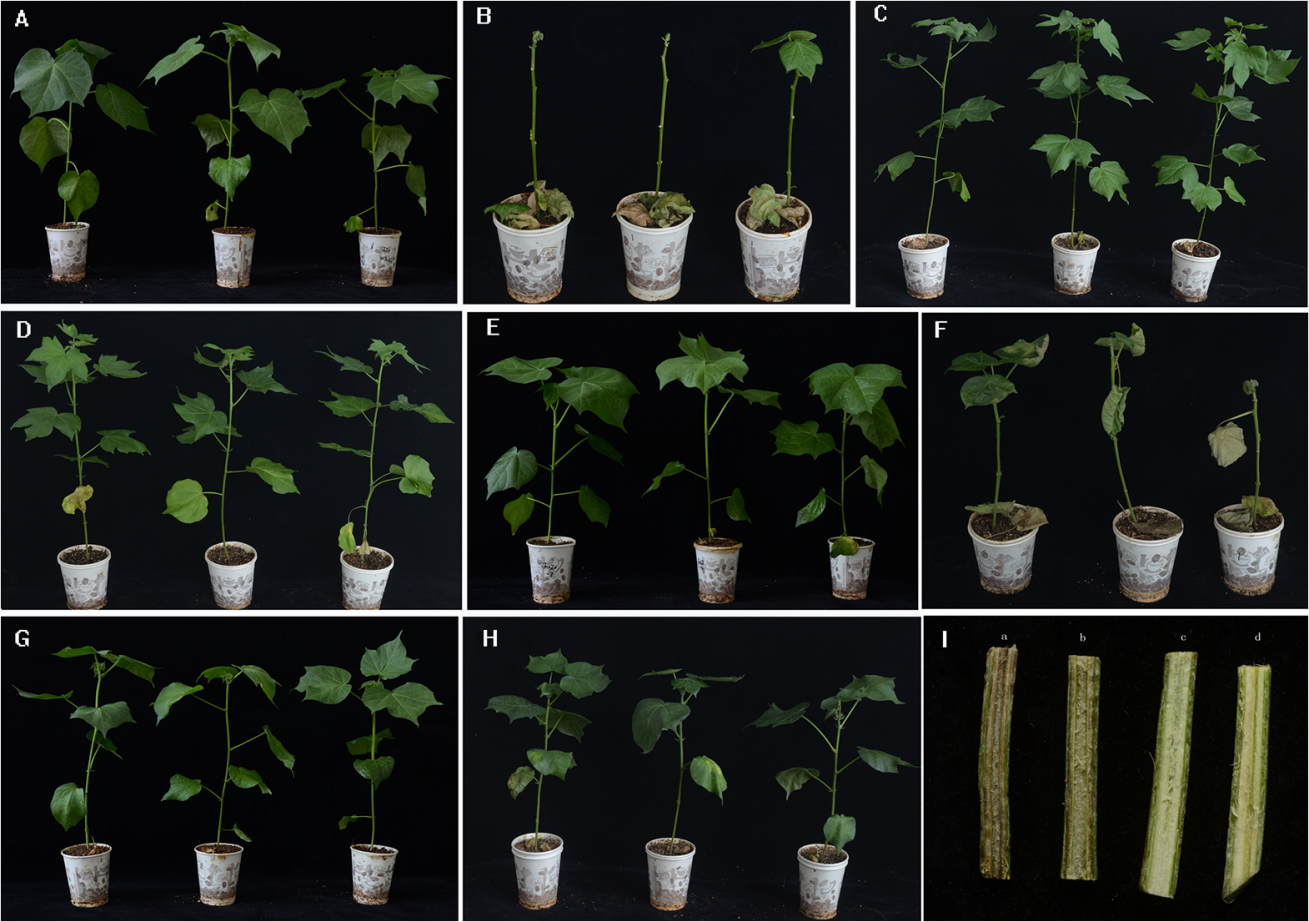
Plant symptom of 35^th^ day inoculated by *Verticillium dahliae* isolates V991. A, Junmian 1 (non-inoculation,Verticillium-sensitive control); B, Junmian 1 (inoculated, severe diseased); C, Shixiya 1 (non-inoculation); D, Shixiya 1 (inoculated, high resistant); E, TM-1 (non-inoculation); F, TM-1 (inoculated, severe diseased); G, S_**1**_ (non-inoculation); S_**1**_, (inoculated, resistant); I, Symptom in the vascular in cotton stems (a, Junmian 1, dark brown vascular tissue; b, TM-1, dark brown vascular tissue; c, Shixiya 1, clear vascular tissue; d, S_**1**_, near clear vascular tissue).

Verticillium wilt (VW) is a destructive disease for cotton, which results into huge loss of lint yield but also deterioration of fiber quality worldwide. Here, we test the resistance against *Verticillium dahlia* of two parents and S_1_ by the strong defoliating isolate V991. *G*. *hirsutum* var. Junmian1 is highly sensitive to VW as control. Verticillium wilt disease index was investigated from the beginning of the 11^th^ day of onset after inoculation to 35^th^ days, the survey once every three days. The results indicate that disease incidence and severity, and its onset of VW were significant different (**Figs 7 and 8**). Junmian 1 and TM-1 were severe and earlier onset of VW than Shixiya 1, while S_1_ was between TM-1 and Shixiya 1. At around 35 days after inoculation, distinct disease symptoms were apparent on all plants inoculated with V991. The disease index (DI) was 100% for Junmian1 and 77.2% for TM-1, but Shixiya1 showed very low DI, only 11.1%. And S_1_ was between TM-1 and Shixiya 1, about 36.7% of DI. The relative resistance index demonstrated that Junmian 1 and TM-1 are susceptible (S) to VW, S_1_ is tolerant (R) and Shixiya1 is resistant (HR) (**Table 9**), implying that Shixiya 1 possesses the potential for improving Upland cotton resistance to VW.

**Table 9:** The results of Junmian1, TM-1, Shixiya 1 and S_1_ by V991 infection.

Materials	Disease index	Relative resistance index	Classification
Junmian1 (CK)	100	50	S
TM-1	77.22±2.07	38.61±1.04	S
Shixiya1	11.67±1.36	5.83±0.68	HR
S_1_	35. 56±2.83	17.78±1.42	R

## Discussion

*G*. *arboreum*, one of the two diploid cultivated cotton species, possesses many favorable genes, such as genes conferring resistance to pests (*Apolygus lucorum*) [8,9] and diseases (infection by *Verticillium dahliae*, *Fusarium oxysporum* vasinfectum and cotton leaf curl virus) [10–13] and tolerance to drought [14]. These traits are much requisite for *G*. *hirsutum*, one of the two tetraploid cultivated cotton species, accounting for >90% of cotton production worldwide.

To transfer favorable genes from *G*. *arboreum* into *G*. *hirsutum*, a prerequisite is overcoming the cross incompatibility that exists between the above two species to obtain interspecific hybrids. For many years, numerous attempts have been made to produce these interspecific hybrids, including ovule and embryo culture studies [17,18,23]. However, it is still quite difficult to obtain these hybrids under *in situ* conditions. A prerequisite for ovule and embryo culture is to ensure that cross-pollinated bolls remain and develop on cotton plants. Thus, during the first three to five days post pollination, the once-daily application of a mixture of the growth regulators NAA (50 mg l^-1^) and GA_3_ (100 mg l^-1^) is necessary to prevent boll shedding [40]. However, we found that the application of GA_3_ (50 mg l^-1^) alone also prevents boll shedding. In addition, the current results reveal that medium containing only one type of plant hormone, Kin, is sufficient for ovule culture, while the addition of IAA and/or GA_3_ to the medium leads to only callus and fiber proliferation. In this study, on the bases of Sacks, Gill and Bajaj, and Thengane et al. [18,20,23], we improved and simplified an embryo rescue technique. Our results proved that MSB2K supplemented with 0.5 mgl^-1^ kinetin and 250 mg^-1^ casein hydrolysate would be an efficient initial medium for rescuing early (3 d after pollination) hybrid embryos.

The obtained triploid hybrids, which contained 39 chromosomes, were still very high sterile due to ploidy barriers that caused aberrant chromosome segregation at meiosis [41]. We also found that there were numerous univalents, trivalents, quadrivalents and hexavalents in the pollen mother cells of the triploids. Therefore, to transfer useful genes from *G*. *arboreum* into *G*. *hirsutum*, the triploid lines should be treated with colchicine to form hexaploid lines, thereby restoring their fertility through chromosome doubling. However, chromosome doubling is not easy to approach even though numerous protocols were reported. In this study, fortunately, we successfully obtained four hexaploid plants using 0.1% colchicines for 24h, all of which set seeds. The result indicated that our protocol would prove to be effective in doubling cotton chromosome sets.

Moreover, S_1_ progenies derived from the hexaploids self-pollinated were assessed for resistance to VW and drought. The preliminary results demonstrated that S_1_ has the potential for transferring resistance genes from *G*. *arboreum* into *G*. *hirsutum* by backcrossing. And these hexaploid plants were also backcrossed with *G*. *hirsutum* (TM-1) and have produced enough backcrossing seeds. These backcross progenies will be consecutively backcrossed with *G*. *hirsutum* for 4~5 times, which would allows us to develop *G*. *arboreum*-introgressed lines with the uniform genetic background of TM-1 by molecular marker-assisted selection. It is well known that linkage drag often exists in interspecific hybridization and leads to a very low rate of cross-over between favorable genes and unfavorable genes from two species particularly having chromosomal structure changes between them, e.g., chromosome translocation existences between *G*. *hirsutum* and *arboreum*. *G*. *arboreum*-introgressed lines, however, have only one or several segments from the donor chromosomes, *G*. *arboreum*, which would eliminate linkage-drag at the most extent. Meanwhile, the genome of *G*. *arboreum* cv Shixiya 1 has been DNA sequenced [26] and that of *G*. *hirsutum* acc. TM-1 (a genetic standard line) is currently being sequenced. Therefore, development of *arboreum*-introgressed lines with the TM-1 uniform genetic background would rapidly and high efficiently bridge the genotype and phenotype gap using Genotyping-by-sequencing (GBS), which would greatly enhance and simplify the mining, isolation, characterization, cloning and use of *G*. *arboreum*-specific desirable genes.
